# Effects of music training in executive function performance in children: A systematic review

**DOI:** 10.3389/fpsyg.2022.968144

**Published:** 2022-08-08

**Authors:** Diego Alejandro Rodriguez-Gomez, Claudia Talero-Gutiérrez

**Affiliations:** Neuroscience Research Group (NeURos), NeuroVitae Center for Neuroscience, School of Medicine and Health Sciences, Universidad del Rosario, Bogotá, Colombia

**Keywords:** executive functions, music training, children, inhibitory control, working memory, cognitive flexibility

## Abstract

Music training has traditionally been a fundamental component of children's education across several cultures. Moreover, music training has been hypothesized to enhance the development of executive functions and improve executive performance in children. In this systematic review, we analyze the available evidence of the effects of music training on executive function performance, evaluated using validated neuropsychologic batteries and classic tasks. To achieve this objective, we performed a systematic search in three databases (PubMed, Ovid MEDLINE, and Scopus) and selected case-control or intervention studies conducted on children with neurotypical development. We analyzed 29 studies that met the inclusion criteria and observed significant heterogeneity among the music interventions and methods for assessing executive functions. The review of the available literature suggests a beneficial effect of music training in core executive function performance, primarily in inhibitory control, and to a lesser extent, in working memory and cognitive flexibility.

## Introduction

History has yet to show one human civilization that does not engage in cultural practices that we would categorize as music. We can only compare the fascinating compulsion to engage with music with basic human needs, such as food and shelter. Whether music is a by-product of other human capacities or evolved for specific adaptive purposes remains unclear (Savage et al., 2021). Nevertheless, music is consistently associated with beneficial properties for human health, and several studies propose a possible benefit of music as adjunctive therapy for cardiovascular and neurologic conditions (Talero-Gutiérrez and Saade-Lemus, 2018). Moreover, hypotheses around music and its role in human life include evocating emotional responses, aiding social development, and increasing academic performance or intelligence (Schellenberg and Weiss, 2013; Sachs et al., 2018; Savage et al., 2021).

From the widely known “Mozart Effect” to including “musical intelligence” in the initial theory of multiple intelligences, there are uncountable theories on the effect of music on cognitive abilities (Jenkins, 2001; Schellenberg and Weiss, 2013; Talero-Gutiérrez and Saade-Lemus, 2018). However, scientific evidence that supports these effects of music mainly exists as anecdotal, observational, or in non-randomized and non-controlled interventional studies. Even in the absence of unequivocal evidence on the impact of music on cognitive development, musical education remains a desirable ability in children and curricular education programs worldwide (Carioti et al., 2019). When evaluating these associations, it may be convenient to point at specific cognitive processes influenced by music rather than assessing cognitive development altogether.

Executive functions (EFs) are a group of mental processes oriented toward goal-directed, purposeful behaviors (Zelazo et al., 1997; Anderson, 2002; Diamond, 2013). Activation of EFs is effortful and requires the recruitment of several brain structures to avoid relying on instinct or intuitive behavior (Diamond, 2013; Cristofori et al., 2019). Different models have proposed a framework for the organization and development of EFs. In one model, there are three core EFs: inhibition or inhibitory control (including behavioral inhibition, selective attention, and cognitive inhibition), working memory (WM), and cognitive flexibility (also called set-shifting and mental flexibility) (Miyake et al., 2000; Lehto et al., 2003). In this model, the three core functions interact to build higher-order EFs: reasoning, problem-solving, and planning (Collins and Koechlin, 2012; Diamond, 2013). More recent models have relied heavily on functional neuroimaging evidence from the prefrontal cortex (PFC). These techniques have revealed functional activation of specialized prefrontal regions when performing different higher-order mental processes (Badre and D'Esposito, 2007; Koechlin and Summerfield, 2007).

Working memory is the ability to retain information while actively working on other mental processes; or inhibiting distraction and interference. Some examples of working memory are holding a question or a comment while engaging in a conversation; or keeping a previous sentence in mind while reading a book (Cowan, 2014; Diamond, 2020). Inhibitory control is the ability to exercise voluntary control over our reactions and behaviors. This EF is critical for avoiding social faux pas and controlling the response to external and internal stimuli (Diamond, 2020). Two subdomains of inhibitory control have been identified and can be evaluated using different tests. Response inhibition is the ability to restrain impulsive or prepotent motor behaviors while attentional inhibition refers to interference control (i.e., the ability to adequately process interfering stimuli) (Tiego et al., 2018). For example, the former subdomain can be evaluated using go/no-go tasks while the latter is usually evaluated with Stroop tasks. In older children, inhibitory control can also be assessed by looking at behavioral measurements of impulsiveness and self-control (Alemán et al., 2017). Lastly, cognitive flexibility, set-shifting, or mental flexibility is the ability to switch between different tasks or mindsets. This EF also includes rapidly and flexibly adapting to sudden change. An example of cognitive flexibility on an everyday basis includes taking an alternative route to a destination when the intended path is unavailable (Diamond, 2020).

In his pivotal work, Piaget observed children as young as 8–12 months purposely reaching hiding objects or using one object as a means to have access to another. This display of intentional, goal-directed behavior was considered indirect evidence of executive control and early development of executive functions (Aguiar and Baillargeon, 2002; Diamond, 2020). Further studies revealed that even younger children (3–3.5 months old) can maintain and update basic information about occluded objects (Aguiar and Baillargeon, 2002). These early insights on the development of working memory have demonstrated that object permanence and early executive control appear before the first year of life. Moreover, several studies have identified that early-life stress is associated with impaired cognitive control during adolescence, supporting the early development of executive functions (Mueller et al., 2010).

Significant improvement in cognitive flexibility and inhibitory control characterizes the late preschool and early school years. Children transition from remarkable rigidity to improved performance in tasks that require impulse control and set-shifting (Munakata et al., 2012; Chevalier et al., 2013, 2015; Diamond, 2020). Nevertheless, preschool children exhibit reactive inhibitory control in response to specific situations, yet they do not develop planning and proactive inhibitory control until around 6–8 years (Munakata et al., 2012; Chevalier et al., 2013). These findings support the theory of the hierarchical development of EFs. In this paradigm, core EFs develop first, and higher-order EFs (such as planning) appear at a later age (Davidson et al., 2006; Shing et al., 2010; Garon et al., 2014).

The quest for understanding executive control and EF development in children has led to multiple hypotheses on whether external stimuli participate in this process. Bilingualism, physical activity, and music education are some examples of interventions proposed to be positive for the development of EFs (Cristofori et al., 2019). Rauscher's classic work in 1993 described an 8–9-point increase in the intelligence coefficient (IQ) score of college students exposed to Mozart's K. 499 sonata (Rauscher et al., 1993; Talero-Gutiérrez and Saade-Lemus, 2018). Although the authors clearly stated that the effect was temporal and observed initially in adults, the public reception of these findings eventually led to the marketing of classical music to promote intellectual development in children. We now know that simply listening to music is not associated with better development of cognitive abilities (Jenkins, 2001; Rauscher and Hinton, 2006; Talero-Gutiérrez and Saade-Lemus, 2018). However, whether music education may affect overall intelligence or specific cognitive processes (such as EFs) is still a field of current research.

In this systematic review, we attempt to evaluate the available evidence on music education's effect on children's executive function development. We hypothesize that music education, not exposure, may be associated with improved domains of EFs and might have a beneficial long-term effect on cognitive development.

## Methods

### Study design

We conducted a systematic review based on the following PICOS question: In children with neurotypical development, is music training compared to other educational or sports intervention, associated with improved executive function performance?

### Search strategy

We searched the literature systematically to identify relevant articles for inclusion in this systematic review. We performed the search on May 5th, 2022, using three databases: PubMed, Ovid MEDLINE, and Scopus. There was no filter by publication date, language, or article type. Table 1 summarizes the terms used in each database. Additionally, we performed a manual search in the references for each included paper to retrieve additional relevant studies.

**Table 1:** Overview of the search strategy, terms, and results in each database.

**Database**	**Search strategy**	**Results**
PubMed	(“Music”[Mesh] OR “Music Therapy”[Mesh]) AND “Child”[Mesh] AND “Executive Function”[Mesh]	11
	“music, executive function, children”	47
Ovid MEDLINE	1—children.mp. or exp Child/	29
	2—executive function.mp. or exp Executive Function/	
	3—exp Music Therapy/ or exp Music/ or music.mp.	
	4–1 and 2 and 3	
Scopus	TITLE-ABS-KEY (children) AND (TITLE-ABS-KEY (music) OR TITLE-ABS-KEY (music AND therapy)) AND TITLE-ABS-KEY (executive AND function)	93

### Eligibility and study selection

We applied the following inclusion criteria to determine whether articles were eligible for this systematic review: (1) Studies conducted on children from 0 to 18 years with neurotypical development and no hearing or visual impairment. (2) Case-control studies or intervention studies with a control group (quasi-experimental and randomized controlled trials). (3) Music education as described in the “music education” subheading in this section. (4) Executive function assessment in any domain described below. We selected articles that used validated neuropsychological batteries or classic tasks. Studies must also include a reproducible scoring system.

Screening for eligibility was performed by both authors simultaneously using Rayyan software based on title, study design, and abstract (Ouzzani et al., 2016). We resolved any disagreements by discussion among the authors.

### Quality assessment

We evaluated each article using the Joanna Briggs Institute (JBI) clinical appraisal tools (https://jbi.global/critical-appraisal-tools). The JBI tools provide a template for qualitative evaluation based on the study design and a checklist of the relevant items to determine the trustworthiness and relevance of the results. Given that this is a qualitative tool, the decision to exclude an article based on the assessment required discussion and unanimous agreement by both authors.

### Music education

We included instrumental and non-instrumental musical interventions. Exposure to music-enriched environments or passive exposure to music (e.g., listening only) was not acceptable for this review. For experimental studies, educational interventions were significant if children received at least 30 min of daily music training for 20 days or an equivalent amount of dedicated training. For case-control studies, we included children with prior music training for a minimum of 3 months.

### Evaluation of executive function

We defined executive functions (EF) as a group of higher-order inter-related processes responsible for purposeful, goal-directed behavior (Anderson, 2002; Zelazo et al., 2003; Diamond, 2013; Cristofori et al., 2019). To select eligible articles and create a data frame for data extraction, we created seven EF domains and assigned each of the tasks or neuropsychologic tests used in the included studies to one of those domains. Most of the evaluations used to assess EFs usually comprise more than one cognitive process at a time. As a result, including a task within a domain simply means that this is the chief process hypothesized to be evaluated by said task. Table 2 presents the seven EF domains included in this review.

**Table 2:** Overview of the executive function (EF) domains and tasks used to evaluate each domain.

**EF domain**	**Tasks**
Cognitive flexibility	Trail making test, Dimensional Change Card Sort (DCCS), Wisconsin Card Sorting Task (WCST), “Peg tapping” task, NEPSY-II subtest: “Animal Sorting”
Working memory	Visuospatial—Matrix span test, Corsi Block test (forward, backward), dot-matrix task, visual pattern span, symbol search, NEPSY-II subtest: “Memory for Designs”
	Verbal—Digit span (forward, backward), color span, word span, updating information task, NEPSY-II subtest: “Sentence repetition”
Inhibitory control	Go/No-Go task, Stroop task, Flanker task, Simon task, Stop-signal task, Matching familiar figures test (MFFT), NEPSY-II subtests: “Statue” and “Inhibition”
Planning and organizing	Tower of Hanoi (ToH), Tower of London (ToL), NEPSY-II subtests: “Tower” and “Clocks”
Selective attention	NEPSY-II subtest: “Auditory attention”
Fluency	NEPSY-II subtest: “Design fluency” and “Verbal fluency,” Phonologic fluency task
Global EF evaluation	“Spin the Pots,” WISC-III and IV batteries, NEPSY-II battery, BRIEF assessment, KBIT battery

*WISC, Wechsler Intelligence Scale for Children; BRIEF, Behavior Rating Inventory of Executive Function; KBIT, Kaufman Brief Intelligence Test*.

### Data extraction and analysis

We extracted the following data from the included articles: study design, population (music and non-music groups), mean age (in months), country, music education type (instrumental o non-instrumental), duration of music education (in weeks), executive function domains, tasks and scoring system, control variables, and main findings. We divided the studies for qualitative analysis purposes using the mean age of each article. The studies were classified as performed in preschoolers (<72 months), school-age children (between 72 and 144 months), and adolescents (more than 144 months). Based on the widely diverse and heterogeneous study designs, we decided to present the results as a narrative review, analyzing the impact of music education in each of the executive function domains.

## Results

### Search results and included studies

**Figure 1:**
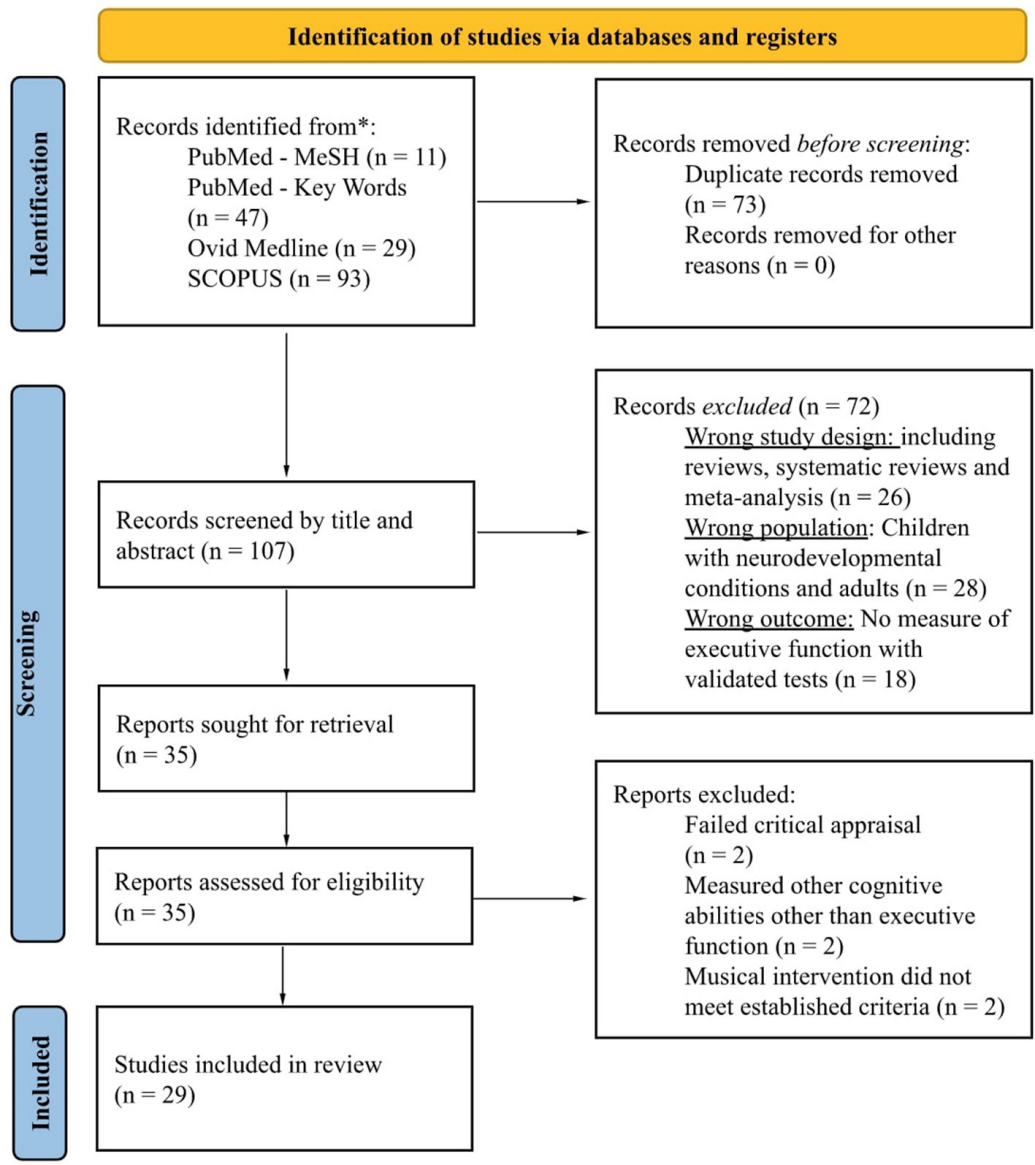
PRISMA flow diagram for the studies included in the systematic review.

We identified a total of 180 records across three databases. No additional studies in the manual search were deemed acceptable for inclusion. The Rayyan software labeled 73 records as possible duplicates. We manually confirmed and excluded these studies from the search. The title and abstract screening featured the 107 remaining articles, 72 of which did not meet inclusion criteria based on the study design, population, or outcome. Lastly, we evaluated full-text eligibility in 35 studies, excluding six of them based on our previously established criteria and JBI appraisal. This systematic review includes 29 records that passed the eligibility process. Figure 1 further specifies the details of the screening process following the PRISMA 2020 Statement (Page et al., 2021).

### Demographic characteristics

Among the 29 included studies, 10 were case-control studies, and 19 were experimental designs (eight quasi-experimental studies and 11 randomized controlled trials). Overall, we identified 2,693 children allocated to music education, either as “cases” in case-control studies or as part of the intervention group in experimental designs. We also identified 2,775 children in the “control” group. The mean age of all children in our systematic review was 103.4 months (8.61 years). Fifteen out of 29 studies (51.7%) were located in the United States (*n* = 6), Germany (*n* = 5), and Canada (*n* = 4). Tables 3, 4 contain key information of all the studies included in this review.

**Table 3:** Overview of the studies evaluating inhibitory control among preschool children.

**Study**	**Intervention and summary of findings**
Moreno et al. (2011)	Significant improvement in Go/No-Go tasks among Canadian children who participated in a high-intensity, 4-week computer-based music education program (*n* = 32) compared to a visual arts program (*n* = 32).
Bugos and DeMarie (2017)	No difference in a day/night Stroop task among 17 preschoolers who participated in a 6-week instrumental and vocal music education program compared to a Lego intervention group (*n* = 17).
Bowmer et al. (2018)	Phase 1: 14 children allocated to non-instrumental 8-week music education demonstrated increased performance in inhibition tasks “Peg tapping” and “Baby Stroop” compared to the control group. In phase 2 (with two music education groups), results were non-significant.
Frischen et al. (2019)	Significant effect of time without group effect in ANOVA analysis comparing the results in NEPSY subtests among a rhythm and pitch training group and a sports intervention group in German preschoolers.
Shen et al. (2019)	Significant group effect in ANOVA analysis comparing 31 Chinese children who participated in a 12-week (150 min/wk) non-instrumental music education program vs. a control group (*n* = 30).
Degé et al. (2022)	Significant improvement in the NEPSY “statue” subtest score (p = 0.02) among 11 German preschoolers who participated in a 14-week music intervention vs. a sports group (*n* = 14).
Bolduc et al. (2021)	Significant improvement in NEPSY “Inhibition” among 50 Canadian preschoolers assigned to a 19-week non-instrumental music intervention compared to a motor intervention (*n* = 52) and control group (*n* = 58).
Kosokabe et al. (2021)	Japanese preschoolers assigned to a music play or a dramatic play program (30 sessions) displayed significantly improved performance in a Go/No-Go task vs. a control group.
Bayanova et al. (2022)	Case-control study evaluating 47 Russian preschoolers that received “extra music classes” with 47 who received regular music education at school. Significantly improved performance in the NEPSY “inhibition” subtest for the “extra music classes” group.

**Table 4:** Summary of all the studies included in this systematic review.

**First author**	**Title**	**Population**	**Intervention**	**EF assessment tools**	**Summary of outcomes**
Degé et al. (2022) (M)	The influence of music training on motoric inhibition in German preschool children	25 Preschoolers in two groups 1. Music intervention group *N* = 11 2. Sports intervention group *N* = 14	Mixed music intervention based on singing, rhythm training, and drumming 20 min, three times a week, for 14 weeks.	“Statue” subtest from NEPSY-II	Significant enhancement in inhibition from pre- to post-test in the music training group compared to the sports control group.
Bayanova et al. (2022) (M)	Difference in executive functions development level between two groups	94 Senior preschoolers in two groups 1. “Extra music classes” group *N* = 47 2. “Regular music classes” control group *N* = 47	“Extra” music classes twice a week for at least 1 month (instrumental and comprehensive music training)	NEPSY-II subtests: “Sentence Repetition,” “Memory for Designs,” “Inhibition,” “Statue,” Dimensional Change Card Sort (DCCS)	Extra music classes group demonstrated improved performance in “sentence repetition,” “DCCS” and “inhibition.” Complete results are summarized in Table 1 of the original paper.
Chen et al. (2022) (M)	The relationship between early musical training and executive functions: Validation of effects of the sensitive period	151 School-age children in two groups 1. Prior music training *N* = 75 2. No prior music training *N* = 76	Music trained group: Instrumental or vocal music training with a minimum of 3 years of experience.	Go/No-Go task, Stroop task, Continuous Performance Task AX-CPT task, Task-switching	The music group showed higher scores in the go/no-go task, lower interference effects in the Stroop task and they outperformed the control group in the AX-CPT task. No significant difference in the task-switching paradigm.
Ilari et al. (2021) (N)	Musical activities, prosocial behaviors, and executive function skills of kindergarten children	103 Kindergarten students in two groups 1. Music intervention group *N* = 51 2. Control group *N* = 52	In-school music classes, mostly non-instrumental training (described in detail in the methods section). 40 min, twice a week, 5 weeks.	DCCS, Spin the pots, prosocial game developed by the authors	No statistically significant difference in prosocial skills, working memory, and inhibition control. Significant pre- to post-test improvement in the music training group compared to the control group.
Frischen et al. (2021) (I)	Music lessons enhance executive functions in 6- to 7-year-old children	94 School-age children in three groups 1. Music training group *N* = 27 2. Visual arts training group *N* = 31 3. Control group *N* = 37	Music/Arts training once per week for 45 min by professional music or visual arts teachers, training was instrument-specific during an 8-month training period with homework. The control group had no intervention.	NEPSY-II subtests: “inhibition,” “auditory attention,” “animal sorting,” “design fluency,” “clocks” and the Working Memory Test Battery subtests: “Matrix,” “Corsi Block”	ANOVA analysis revealed significant improvement with music intervention in inhibition and visuospatial working memory in the pre- to post-test comparison against the other groups. There was also significant improvement in pre- to post-test scores within the music group on the selective attention EF, however, post-test scores compared to other groups were non-significant.
Putkinen et al. (2021) (C)	Faster maturation of selective attention in musically trained children and adolescents: Converging behavioral and event-related potential evidence	80 Late school-age children and adolescents in two groups 1. Prior music training *N* = 44 2. No prior music training *N* = 36	Music group had been taking lessons starting at age 7. Control group had no formal music training.	NEPSY-II subtest: inhibition	Faster completion times in the music group, no statistically significant differences in the executive function tasks measured
Bolduc et al. (2021) (N)	The impact of music training on inhibition control, phonological processing, and motor skills in kindergarteners: A randomized control trial	160 Preschoolers assigned to three groups 1. Music intervention group *N* = 50 2. Motor training group *N* = 52 3. Control group *N* = 58	Motor and music interventions with six themes covered in 19 weeks, twice a week, 40 min each (detailed description in the methods)	NEPSY-II subtest: inhibition-inhibition (INI) task	The music intervention was significantly associated with improved performance in the INI task in three tests of the ANOVA (conditions, time, time * conditions)
Frischen et al. (2019) (N)	Comparing the effects of rhythm-based music training and pitch-based music training on executive functions in preschoolers	76 Preschoolers in three groups 1. Pitch training group *N* = 27 2. Rhythm training group *N* = 26 3. Sports training group *N* = 23	Non-instrumental pitch training and rhythm training. 20 min of training three times a week for 20 weeks.	NEPSY-II subtest: “Statue.” DCCS standard and border versions, Matrix span test, Corsi Block test	Significant time effect for all the evaluated executive functions. A significant effect of group was only observed when comparing inhibitory control between rhythm training and the sports group.
Bowmer et al. (2018) (N)	Investigating the impact of a musical intervention on preschool children's executive function	Phase 1 Three groups: (A) Music *N* = 14, (B) and (C) No Music *N* = 25 Phase 2 Three groups: (A) Music (16-weeks) *N* = 14, (B) Music (8-weeks) *N* = 15, (C) Visual arts *N* = 12	Group A initiated music intervention in phase 1, group B in phase 2. The intervention were non-instrumental lessons. 40 min weekly, 8 weeks each phase, two phases.	Peg Tapping, Truck, ToL, DCCS, Baby Stroop, Spin the pots, BRIEF-P	Phase 1 showed Group A (music) to have significantly improved performance in planning and inhibition skills. Phase 2 found no significant difference in performance between the groups. However, the music intervention was nearly significant for improved performance in the peg tapping task (*p* = 0.06).
Jaschke et al. (2018) (I)	Longitudinal analysis of music education on executive functions in primary school children	147 School-age children in four groups 1. Music with prior knowledge *N* = 38 2. Music with no prior knowledge *N* = 42 3. Visual arts *N* = 29 4. No arts control *N* = 37	Musical lessons, instrument-based training receiving 1–2 h lessons weekly as part of the school curriculum. Visual arts: General lessons in painting, sculpting, and art history.	Tower of London (scoring method in the supplementary material—designed by the authors), Klingberg memory task with dot matrix, scoring was designed by the authors. Inhibition: Go/No-Go task	WM: Significant increase in the visual arts group compared to the no arts and both music groups. Planning: significant increase in the two music groups compared to visual arts and control. Inhibition: Significant group × time interaction in the two music groups.
Guo et al. (2018) (I)	Improved digit span in children after a 6-week intervention of playing a musical instrument: An exploratory randomized controlled trial	40 School-age children in two groups 1. Music intervention *N* = 20 2. Control *N* = 20	Instrumental training with keyboard harmonica. 12 sessions in 6 weeks (25 min/session)	Digit span test, go/no-go test, WISC-IV digit symbol	Significant improvement in the Digit Span test (especially in the Digit Span Backward) compared to the control group.
Herrero and Carriedo (2018) (C)	Differences in updating processes between musicians and non-musicians from late childhood to adolescence	138 Late school-age children and adolescents in 4 groups 1. 3rd−4th grade, *N* = 37 with 3 years of music training 2. 3rd−4th grade, *N* = 37 with no musical training 3. 9th−10th grade, *N* = 32 with 7 years of music training 4. 9th−10th grade, *N* = 32 with no music training	Musicians had been exposed to music theory and instrument interpretation and composition in a traditional conservatory (at least 3 years)	WM: Updating information task as described by Beni and Palladino (2004). 24 lists each with 12 auditory words in a standardized computer software	Musicians outperformed the control group in all experimental conditions for the proportion of intrusion errors but not in the recall of critical words (inhibitory and maintenance processes and resistance to proactive interference)
Bugos and DeMarie (2017) (I)	The effects of a short-term music program on preschool children's executive functions	34 Preschool children in two groups 1. Music intervention *N* = 17 2. Lego construction (control) *N* = 17	Instrument-based training: Electronic and acoustic instruments with vocal development exercises and improvisational activities. Six weeks of training with two 45-min weekly classes.	Day/Night Stroop test. Matching familiar figures test (MFFT)	Significant time and group effect in the MFFT with fewer errors committed in the group that received the music intervention. No effect was seen in the Stroop task.
Joret et al. (2017) (C)	Cognitive inhibitory control in children following early childhood music education	61 School-age children in two groups 1. Music trained since 5 years old *N* = 30 2. Non-music trained *N* = 31	Musicians had been exposed to music theory and instrument interpretation and composition in a traditional conservatory starting at age 5 or younger	Automated Simon task (Explanation of congruent—incongruent tests can be found in the original paper)	Significant association between music training, congruency testing and reaction times (RT). Musicians outperformed the control groups in tests that were non-congruent.
Holochwost et al. (2017) (I)	Music education, academic achievement, and executive functions	265 School-age children in two groups 1. Music intervention *N* = 135 2. Control group *N* = 130	Intensive course of music during the academic years of 2010-2013 inspired by El Sistema. The program ran for 39 weeks each year and consisted of 2 hours/day with 40 min instrument instruction and 40 min rehearsal.	Tower of London (ToL), Wisconsin Card Sorting Task, go/no-go task, Stroop task, trail-making task, flanker task.	Significant improvement in the flanker test, card-sorting tasks, go/no-go test, memory span, and reaction times (RT) for the Stroop test. No significant differences were observed in the ToL, trail-making, and Corsi tasks.
Saarikivi et al. (2016) (I)	Cognitive flexibility modulates maturation and music-training related changes in neural sound discrimination	90 Late school-age children and adolescents in two groups 1. Prior music training starting around age 7 years *N* = 43 2. No prior music training *N* = 47	Instrumental music training starting around 7 years. Mean starting age 6.5 yrs and mean 3.07 yrs of training at the time of measurement	NEPSY-II test battery: Inhibition, verbal fluency, and trail-making test subtests (part B). Backward digit span test from WISC-IV	Musically trained participants had shorter completion times than non-trained participants in naming, inhibition, and set-shifting tasks. There were no group differences in performance.
Roden et al. (2014) (I)	Does music training enhance working memory performance? Findings from a quasi-experimental longitudinal study	50 School-age children in two groups 1. Music training program *N* = 25 2. Natural science training program *N* = 25	Instrumental weekly music lessons (45 min) and practice at home. 18-month study period with multiple neuropsychologic testing	General: Counting span test, complex span test, color span backward test. WM: Corsi block test, matrix span	No significant differences in the Matrix Span test or the Corsi Block Test. Significant group × time interaction in the counting span test and complex span test. No significant interactions in the color span backward.
Degé et al. (2011) (C)	Music lessons and intelligence: A relation mediated by executive functions	90 School-age children, different degrees of music training 1. No music lessons *N* = 29 2. 1–4 years of music training *N* = 45 3. More than 4 years of music training *N* = 16	Case-control study. Prior music training was assessed using a questionnaire answered by the parents to evaluate the degree of musical training and the number of instruments.	NEPSY-II: Animal sorting, Auditory attention, Clocks, Inhibition, Design fluency	Positive moderate correlation between duration of music lessons and the different executive functions. Specific testing for executive function revealed mediation effects of selective attention and inhibition.
Moreno et al. (2011) (N)	Short-term music training enhances verbal intelligence and executive function.	64 Preschoolers in two groups 1. Music intervention *N* = 32 2. Visual arts intervention *N* = 32	Non-instrumental, computer-based, music education and visual arts programs. 2 daily 1-hour sessions, 5 days a week, 4 weeks.	WPPSI-III (intelligence, verbal ability, spatial ability), go/no-go test	In vocabulary and verbal intelligence the music intervention was associated with increased raw vocabulary score. This finding was also replicated in the go/no-go trials.
Janus et al. (2016) (N)	Effects of short-term music and second-language training on executive control.	57 Monolingual preschoolers in two groups 1. Music training program *N* = 29 2. French education program *N* = 28	Non-instrumental, computer-based music education and french learning program. 3 hours a day with 1-hour breaks during 20 days. Music training was based on rhythm, pitch, melody, voice, and basic musical concepts.	Corsi blocks, verbal fluency, sentence judgment (as described by authors), visual search, word span	Word span: French outperformed Music. Corsi block: No difference. Verbal fluency: Both groups improved. Sentence judgment: Better performance on anomalous sentences. Music did not outperform french in any setting.
Schellenberg (2011) (C)	Examining the association between music lessons and intelligence.	106 School-age children in two groups	Prior music training (at least two years) 1. Prior music training *N* = 50 2. No prior music training *N* = 56	Tower of Hanoi, WCST, Stroop test, Phonologic and semantic fluency, Digit Span	The effect of music training on executive function was non-significant.
Zuk et al. (2014) (C)	Behavioral and neural correlates of executive functioning in musicians and non-musicians.	27 School-age children in two groups 1. Prior music training *N* = 15 2. No prior music training *N* = 12	At least 2 yrs of music training (instrument-based) with private lessons.	Trail-making test, verbal fluency, color-word interference, digit span backward, coding subtests WAIS, Kaufman KBIT	Children in the music group had a better performance in coding, verbal fluency, design fluency, and trail-making test. There was no significant difference in the Stroop or the WM test.
Sachs et al. (2017) (M)	Increased engagement of the cognitive control network associated with music training in children during an fMRI Stroop task.	56 School-age children in three groups 1. Music intervention group *N* = 18 2. Sports intervention group *N* = 18 3. Control group *N* = 20	Youth Orchestra of Los Angeles: 7 weekly hours of music learning divided into string instruments, choir, and musicianship.	WASI-II, digit span, block design, matrix reasoning, Stroop task, “hearts and flowers,” flanker fish task	No significant differences in behavioral performance in any of the tests evaluated.
Nie et al. (2022) (N)	Effects of music training on the auditory working memory of chinese-speaking school-aged children: A longitudinal intervention study.	110 School-age children in three groups 1. Music intervention group *N* = 34 2. Language education group *N* = 46 3. Control group *N* = 30	Music intervention 1 hour daily, 5 days a week for a year using the Kodaly method (non-instrumental)	WISC-IV: Digit span test (forward and backward), block design, and vocabulary.	The musically trained group showed significant superiority compared to the control group in the DS backward performance only.
Hennessy et al. (2019) (M)	Effects of music training on inhibitory control and associated neural networks in school-aged children: A longitudinal study.	88 School-age children were randomized into three groups At the 4-year follow up 60 children remained in study 1. Music intervention group *N* = 28 2. Sports intervention group *N* = 29 3. Control group *N* = 31	Youth Orchestra of Los Angeles: 7 weekly hours of music learning divided into string instruments, choir, and musicianship.	WASI-II, digit span, block design, matrix reasoning, Stroop task, “hearts and flowers,” flanker fish task	No significant differences in the performance on behavioral tasks. However, in a delayed gratification task they did find that the music group tends to choose larger rewards.
Park et al. (2015) (N)	A preliminary study of the effects of an arts education program on executive function, behavior, and brain structure in a sample of nonclinical school-aged children.	29 School-age children in two groups 1. Musical arts intervention group *N* = 14 2. Comprehensive arts intervention group *N* = 15	15-week intervention with two types of arts: comprehensive dance, recreations, and a music arts program. 2 hours per session for 15 sessions	Wisconsin Card Sorting test	Significant improvement on the WCST during the study. However, when analyzing each group, only the comprehensive arts group was found to be statistically significant.
Kosokabe et al. (2021) (N)	Self-directed dramatic and music play programs enhance executive function in Japanese children.	218 Preschool children assigned to 3 groups 1. Music play program *N* = 92 2. Dramatic play program *N* = 51 3. Control group *N* = 75	Music play program created based on the principles of Orff-Schulwerk. The main activities of the music play program included six units, and children experienced each unit five times, resulting in 30 lessons in total.	DCCS, “Hand game”—Go/NoGo, Backward digit and word span	Significant improvement in the groups of dramatic play and music play programs in the working memory and inhibitory control tests compared to the control group.
Alemán et al. (2017) (I)	The effects of musical training on child development: A randomized trial of El Sistema in Venezuela.	2914 School-age children in two groups (ITT analysis) 1. Early admission (2012) to music program *N* = 1480 Only 794 participated in the intervention 2. Delayed admission (2013) to music program *N* = 1434 Only 208 participated in the intervention	El Sistema: In the initial year of participation, school-aged children receive instruction in both an instrument and choral singing. Teacher-led musical instruction occurs several times per week. The instruction takes place in a full ensemble.	Go/No-go, Flanker task, Delay discount, Tower of London, Score forward and backward	There was a significant improvement in self-control in the intervention vs. control groups (assessed by guardian-reported questionnaires) however, there were no significant findings in any of the executive functions evaluated on the sample as a whole. In the sub-group analysis, they did find significant improvement in the go/no-go task among older children (10 to 14 years)
Shen et al. (2019) (N)	Sustained effect of music training on the enhancement of executive function in preschool children.	61 Preschool children in two groups 1. Music intervention group *N* = 30 2. Control group *N* = 31	Combination of motor, perceptual, and cognitive tasks, including training in rhythm, pitch, melody, voice, and basic musical concepts. 45 min each, 5 days a week, for 12 weeks.	Day/Night Stroop, DCCS, Dot matrix test, backward digit span	Significant improvement in all four tests of EF when comparing group × effect interaction in ANOVA.

### Music education and other interventions

We identified a heterogeneous array of music interventions. These were classified into four groups: instrument-based music training (I), non-instrumental music training (N), mixed music education programs (M), and prior classic or private music lessons (C). Interventions were considered to be instrument-based when more than half of the lessons were instrumental; eight studies satisfied this criterion. In most of these studies, children were able to select the instrument of their preference; however, in other studies the instrument was defined for all the participants (Guo et al., 2018). Non-instrumental programs (*n* = 10 studies) mostly included lessons based on rhythm, pitch, or vocal training. Some of these interventions were structured around traditional strategies for music education such as the Kodály method.

Five studies were classified as having mixed music education programs that included instrument-based lessons and non-instrumental training. Moreover, we identified six case-control studies evaluating prior classic or private music training. Classic music training was defined as participating in conservatories or orchestra-based programs. Most music training in this group is hypothesized to be instrumental, however, since music training in these cases was not controlled within an experimental study design, we decided to classify them separately. Control interventions included visual arts, sports, and second-language programs. Table 4 contains detailed information on the interventions in each study and a letter indicating the group to which they were assigned.

### Executive function assessment

The assessment tools used to evaluate the EF domains were classic neuropsychologic tasks and validated batteries such as NEPSY-II. Table 2 summarizes the tests used in the assessment of each EF domain. The most frequently assessed EF domains were the three core executive functions: inhibitory control (*n* = 23, 79.3%), working memory (*n* = 19, 65.5%), and cognitive flexibility (*n* = 17, 58.6%). The studies also examined fluency (*n* = 7), planning and organization (*n* = 6), and selective attention (*n* = 5). Lastly, 12 out of the 29 articles (41.3%) included an overall evaluation of executive control.

### Executive function domains

#### Inhibitory control

Twenty-three out of 29 studies investigated the influence of music education on inhibitory control. Preschoolers (*n* = 9), school-age children (*n* = 12), and adolescents (*n* = 2) participated in these studies. Studies with preschool samples used one or more of the following tasks: Stroop, Go/No-Go, and NEPSY “inhibition” or “statue” subtests (Moreno et al., 2011; Bugos and DeMarie, 2017; Bowmer et al., 2018; Frischen et al., 2019; Shen et al., 2019; Bolduc et al., 2021; Kosokabe et al., 2021; Bayanova et al., 2022; Degé et al., 2022). Musical education at this age was primarily non-instrumental and centered on rhythm or pitch training. However, some studies used structured interventions such as the Orff method and music play programs; others included instrument-based education. The intensity of the music education program ranged from 40 min once a week to 2 h daily every weekday. Control variables included in these studies were age, school year, sex, parental education, and prior music training. Six of nine studies on preschoolers identified a significant improvement in inhibitory control. Table 3 summarizes the most relevant findings of these studies.

We identified 12 studies in school-age children, five case-control studies with prior instrumental music learning, three studies with an instrument-based music education program, and four orchestra-based interventions. Six studies observed significantly improved performance in inhibition tasks using the Go/No-Go, Stroop, Simon, and the NEPSY tests (Degé et al., 2011; Schellenberg, 2011; Zuk et al., 2014; Alemán et al., 2017; Holochwost et al., 2017; Joret et al., 2017; Sachs et al., 2017; Guo et al., 2018; Jaschke et al., 2018; Hennessy et al., 2019; Frischen et al., 2021; Chen et al., 2022). Table 4 contains detailed information for each article. In adolescents, the studies by Putniken and Saarkivi failed to identify any improvement in inhibitory control measurements among those with prior music training. Nevertheless, the completion time for the tests was significantly faster in the music-trained groups (Saarikivi et al., 2016; Putkinen et al., 2021).

The largest study in this systematic review evaluated 2,914 school-age children in Venezuela in a year-long, high-intensity, orchestra-based music education program (*n* = 1,480) and a control group (*n* = 1,434) (Alemán et al., 2017). This study failed to identify a significant difference in executive function testing among the groups. Comparably, the article by Sachs et al. (2017) evaluated children who participated in the Youth Orchestra of Los Angeles and those who did not. This study failed to identify significant associations between orchestra-based music training and executive function performance. Nevertheless, a similar study design by Hennessy et al. (2019) identified improved accuracy in the flanker task after 3–4 years of music training, as well as improved performance in a delayed gratification task.

Lastly, the evaluation of self-control and impulsiveness using guardian-reported questionnaires in the study by Alemán et al. (2017) identified a significant improvement throughout the music education program. Several variables such as the length of the interventions and the tasks used to evaluate executive performance may explain the heterogeneity of these results. For instance, Sachs et al. (2017) and Hennessy et al. (2019) measured inhibitory control using the Stroop and Flanker fish tasks while Alemán et al. (2017) used a Go/No-Go task. Moreover, Alemán et al. (2017) also evaluated self-control and impulsiveness through a questionnaire, which are intrinsically linked to the appropriate development of inhibitory control in older children.

#### Working memory

Neuropsychologic testing divides the evaluation of working memory (WM) into visuospatial and verbal WM. As a result, we extracted data from the studies in two separate subdomains for WM. Overall, we identified 19 studies that evaluated at least one component of WM (15 studies evaluated visuospatial WM and 13 verbal WM). WM was evaluated across all age groups: eight studies included preschoolers, nine school-age children, and two adolescents. The most frequently used instruments to assess visuospatial WM were the Matrix span test, Corsi block test, and Dot-matrix task. The studies mostly used digit and word span (forward and backward) tasks to evaluate the verbal WM. Detailed information for each of the studies included in this domain is available on Table 4.

Out of the eight studies on preschool children, only three showed significant improvement in a WM subdomain. Bayanova et al. (2022) evaluated several EFs using NEPSY-II subtests in a Russian preschool sample by comparing children who received “extra music classes” (*n* = 47) to those that did not (*n* = 47). The results demonstrated significantly improved performance in the “Sentence repetition,” “DCCS,” and “Inhibition” subtests, which evaluate verbal WM, cognitive flexibility, and inhibitory control, respectively. Kosokabe et al. (2021) also identified a significant association between music training (music play program based on the principles of Orff-Schulwerk) and EFs. In this study, children who participated in the music play and dramatic play programs had significantly better performance in the Go/No-Go task (inhibitory control) and the Backwards digit and word span tests (verbal WM).

Guo et al. (2018) allocated 40 Japanese school-age children to either a 6-week harmonica lessons program (*n* = 20) or a control intervention (*n* = 20). After the music training program ended, the results showed a significant group × time interaction (*p* = 0.015) in the digit span backward (DSB) performance, suggesting an improved verbal working memory with harmonica training. Comparably, Nie et al. (2022) also identified a significant improvement in the DSB performance (*p* < 0.001) after a year-long music intervention using the non-instrumental; Hungarian, Kódaly method. Neither of these RCTs observed improved performance in visuospatial WM or forward digit/word tasks.

The only study to identify improved performance in visuospatial WM was that by Frischen et al. (2021) in a German sample (*n* = 94, mean age: 78.67 months). In this RCT, 25 children participated in a music arts program once per week for 45 min for 8 months. Neuropsychologic testing included several NEPSY-II and the Working Memory Test Battery subtests: “Matrix” and “Corsi block.” ANOVA analysis revealed a significant group effect in inhibitory control and visuospatial working memory subtests. Other studies in preschoolers and school-age children did not identify differences in WM tests between groups exposed to a music education program and those that participated in other interventions (Moreno et al., 2011; Roden et al., 2014; Janus et al., 2016; Bowmer et al., 2018; Herrero and Carriedo, 2018; Jaschke et al., 2018; Frischen et al., 2019).

In the study by Janus et al. (2016), children who participated in education programs for French as a second language outperformed those who received music training in all behavioral measurements of executive function. Similarly, Jaschke et al. (2018) allocated 147 school-age children from a Netherlands sample to music training, visual arts training, or no arts program. Visuospatial WM significantly improved in the visual arts group compared to other interventions. In Finnish adolescents, two case-control studies failed to identify a significant effect of music training on WM performance using NEPSY tests (Saarikivi et al., 2016; Putkinen et al., 2021).

#### Cognitive flexibility

Cognitive flexibility is one of the three core EFs, and several authors hypothesize that music training has a positive influence on its development. We identified 17 studies that evaluated cognitive flexibility using the trail-making test, DCCS, WCST, or the NEPSY-II subtest: “Animal Sorting.” The studies included preschoolers (*n* = 6), school-age children (*n* = 9), and adolescents (*n* = 1). Detailed information for each of the studies included in this domain is available on Table 4.

Three studies in preschoolers found statistically significant improvement in cognitive flexibility measured using the DCCS task. Ilari et al. (2021) used a quasi-experimental design to allocate 51 preschoolers to a 5-week, in-school music classes and 52 children to a collective non-musical education program. They evaluated several EFs and identified significantly improved performance in the DCCS task in the music education group. After adjusting for age, sex, and baseline cognitive evaluation, post-test results remained significant. Shen et al. (2019) replicated similar findings in a Chinese population (*n* = 61, mean age: 50.86 months) allocated to either a 12-week non-instrumental music education program (*n* = 30) or a control group (*n* = 31). ANOVA analysis found a significant group (*p* < 0.01) and group × time (*p* < 0.05) interactions in the DCCS score for cognitive flexibility. This study also identified improved performance in the dot-matrix task (visuospatial WM), backward digit span (verbal WM), and Stropp task (inhibitory control). Moreover, they also found that the improvement induced by music education persisted 12 weeks after the intervention.

Zuk et al. (2014) compared 15 school-age children with at least 2 years of instrument-based music training with 12 children without prior music education. Children in the music training group had significantly improved results in the trail-making test compared to the control group (*p* = 0.026). Similarly, in a RCT, Holochwost et al. (2017) compared 135 children enrolled in an intensive, instrument-based music course inspired by “El Sistema” with 130 children in a control group. This study found that the music education program was associated with fewer errors in the card-sorting tasks. After adjustment for baseline performance, the music intervention had a greater effect size on the high-performance children.

Despite these positive results, several other studies did not find significant associations between music education and cognitive flexibility tests. The article by Saarikivi et al. (2016) measured cognitive flexibility using the trail-making test from NEPSY-II among a case-control population of Finnish adolescents. The results did not identify a significant group effect interaction in ANOVA, yet the completion time for music-trained children was faster. Comparably, 10 studies (three in preschoolers and seven in school-age children) did not identify a significant association between music training and performance in card-sorting tasks or NEPSY subtests (Degé et al., 2011; Schellenberg, 2011; Park et al., 2015; Sachs et al., 2017; Frischen et al., 2019; Kosokabe et al., 2021; Bayanova et al., 2022; Chen et al., 2022). Once again, the largest study in our review did not identify positive results in the cognitive flexibility tasks among 1,480 children who participated in a year-long, instrument-based education program (Alemán et al., 2017).

#### Fluency

There is an ongoing debate on whether verbal fluency should be considered a language component or an executive function. Some authors have found that the results in EFs assessments do not correlate with those obtained in fluency tests (Whiteside et al., 2016). As a result, we decided to consider fluency a different EF domain that requires separate analysis. Seven articles evaluated verbal or design fluency using either the NEPSY-II subtests or the phonologic fluency task. A total of 572 children (*n* = 261 allocated to music education) participated in the studies included in this domain. The mean age was 110.70 months (9.2 years), and in five of the studies, participants were school-age children (Degé et al., 2011; Schellenberg, 2011; Zuk et al., 2014; Janus et al., 2016; Saarikivi et al., 2016; Frischen et al., 2021; Nie et al., 2022).

In a case-control study, Zuk et al. (2014) evaluated 27 school-aged children, 15 of which received at least 2 years of instrument-based music training. This study found significantly improved scores in the “Verbal Fluency” subtest of the DKEFS battery (*p* = 0.016). However, the remaining studies in this domain did not identify a significant association between music education and fluency test results. Two randomized controlled trials (RCTs) from a German and a Chinese population used subtests from NEPSY and WISC-IV to assess fluency after an instrumental and non-instrumental intervention. Both RCTs failed to identify a significant association between music training and fluency evaluations (Frischen et al., 2021; Nie et al., 2022). Moreover, in a preschool sample, Janus et al. (2016) found that learning French as a second language was associated with improved verbal fluency vs. a non-instrumental, computerized; music education program.

#### Selective attention

Diamond and other authors consider selective attention a subdomain of inhibitory control and not a core executive function per se (Diamond, 2013; Cristofori et al., 2019). However, Grinspun and Veer consider selective attention a separate cognitive process that participates in the development of inhibitory control (Veer et al., 2017; Grinspun et al., 2020). Therefore, we included a domain for selective attention, acknowledging the close relationship that this EF domain has with inhibitory control. Four studies evaluated the effects of music education on selective attention using the “Auditory Attention” subtest of NEPSY-II and the “Peg tapping” task (Degé et al., 2011; Bowmer et al., 2018; Frischen et al., 2021; Putkinen et al., 2021). A total of 303 children (*n* = 159 allocated to the music intervention) participated in these studies. Studies included preschoolers (*n* = 1), adolescents (*n* = 1) and school-age children (*n* = 2); mean age was 116 months (9.66 years).

Bowmer et al. (2018) identified improved performance in the “Peg tapping” task among 29 preschoolers that participated in a non-instrumental music intervention for 8 weeks (*n* = 15) and 16 weeks (*n* = 14) compared to 12 children that participated in a visual arts education program. However, despite the positive findings of this study, the results were barely non-significant (*p* = 0.06). Similarly, Putkinen et al. (2021) did not identify a significant association between prior music education and selective attention performance (NEPSY subtests) in Finnish adolescents.

In contrast, Degé et al. (2011) identified a significant association between school-age children with prior music education and selective attention performance in NEPSY subtests. This case-control study included 61 children with parent-reported music education and 29 age-matched controls. The results demonstrated a moderately positive correlation between duration of music lessons, selective attention, and IQ; after adjusting for sex, parental education, and family income. In a RCT, Frischen et al. (2021) identified a significant improvement in selective attention pre- to post-test scores after participating in an 8-month music education program for German school-age children. Nevertheless, the study did not identify improvement in post-test scores when comparing the music group to a visual arts group and a control (no intervention) group.

#### Planning and organization

Six studies evaluated the Planning and Organization (P&O) domain using either the Tower of London (ToL), Tower of Hanoi (ToH), or the NEPSY subtests “Tower” and “Clocks.” These studies included 3,508 children (*n* = 1,780 allocated to the music education groups). However, 2,914 children participated in one study (Alemán et al., 2017). Studies included school-age children (*n* = 5) and preschoolers (n =1); the mean age was 113.48 months (Degé et al., 2011; Schellenberg, 2011; Holochwost et al., 2017; Bowmer et al., 2018; Frischen et al., 2021).

None of the studies in the school-age children (*n* = 5) category found a significant association between participating in a music education program or having prior music education and the P&O domain performance (Degé et al., 2011; Schellenberg, 2011; Alemán et al., 2017; Holochwost et al., 2017; Frischen et al., 2021). However, in the previously described study by Bowmer et al. (2018), there was a significant improvement between the preschoolers who received non-instrumental music education for 8 weeks (*n* = 15) and the control group. This effect was observed only in phase 1 of the study, consisting of one music intervention group and two control groups. In phase 2 (with two music intervention groups), there were no significant associations between music education and P&O test results.

## Discussion

The music interventions used in this review are heterogeneous. There is no consensus on the adequate intensity for a music education program to have a maximal impact on cognitive function. Consequently, the interventions in these studies ranged from less than an hour per week to twice-daily hour-long sessions on weekdays. Similarly, the music education programs lasted from 3 weeks to a year, and in case-control studies, some children received music training for several years prior to measuring executive functions.

We also observed heterogeneity among the different strategies to deliver music education. Non-instrumental interventions included programs that emphasize pitch and rhythm training, musical play programs, and structured interventions such as those using the Kódaly method. Instrumental interventions included different musical instruments and varied from private at-home lessons to orchestra-based programs. In particular, various studies referenced and used the “El Sistema” orchestra-based program in Venezuela as guidance to develop their interventions (Alemán et al., 2017; Sachs et al., 2017; Hennessy et al., 2019).

Determining whether one type of music education outperforms other music-based strategies remains unclear. We did not consistently identify an intervention that yielded better outcomes in EF performance compared to others. We did hypothesize that consistent with popular beliefs, instrumental music education and orchestra-based music programs would be associated with increased performance in EFs. Surprisingly, we observed minimal-to-no improvement in at least three large RCTs. The study by Alemán et al. was conducted in the “El Sistema” program in Venezuela, while the studies by Sachs and Hennessy included a sample that participated in the Los Angeles Youth Orchestra, inspired by the Venezuelan music education program (Alemán et al., 2017; Sachs et al., 2017; Hennessy et al., 2019).

We did identify evidence supporting an effect of music training on inhibitory control, particularly among preschool-age children. In the nine studies that evaluated inhibitory control in this population, six identified significantly improved performance after music intervention. Music training requires children to pay appropriate attention to sensory stimuli with different characteristics and to integrate multiple stimuli into a rhythm or a melody (Shen et al., 2019). The ability to identify, follow and recreate a rhythm also requires impulse control and authors hypothesize that it could improve inhibition as an EF (Joret et al., 2017). Furthermore, in the study by Degé et al. (2022), the authors identified a larger effect size with an instrumental, active music training program. They hypothesize this effect to be larger due to the nature of the music intervention, suggesting that learning how to play music may have a greater impact on inhibitory control compared to non-instrumental interventions.

Only half of the studies in school-age children identified a significant effect of music training in inhibitory control. Although these studies mostly included instrument-based music programs, one possible explanation is that the tests used to evaluate inhibitory control may be more easily solved by children in this age group compared to preschoolers. Moreover, EFs develop rapidly during the first 3-to-5 years and then continue to evolve during the school years and adolescence to reach an adult-level performance (Best and Miller, 2010). The fact that most articles on preschool-aged children support the effect of music training on inhibitory control probably reflects an age-dependent benefit of music in the development of EFs. In line with this hypothesis, we did not observe improved performance in inhibitory control in the studies performed on adolescents. However, adolescents exposed to music training were able to complete the test faster, with a similar amount of errors (Saarikivi et al., 2016; Putkinen et al., 2021).

Evidence of music education in EFs other than inhibitory control was incongruous. The studies did not consistently identify a significant improvement in EF task performance for the remaining core EF domains (working memory and cognitive flexibility). In the working memory domain, we identified studies that observed improved performance in verbal working memory after music training. However, non-musical interventions such as visual arts training and learning a second language outperformed music in the visuospatial and verbal WM subdomains (Janus et al., 2016; Frischen et al., 2021). The case for cognitive flexibility was similar: although some studies did identify significant improvement in sorting tasks, primarily among preschoolers, the larger studies in school-age children and adolescents found no associations (Zuk et al., 2014; Shen et al., 2019; Ilari et al., 2021).

On the other hand, the relation between music education and non-core EF domains is even weaker. Only one study identified improved performance in planning tasks after music training, and those results became non-significant when replicating the findings with a second music education group (Bowmer et al., 2018). Similarly, whether we consider fluency to be an EF or a cognitive ability related to language development, the results by Janus et al. (2016) suggest that the effect of music education on verbal fluency is probably negligible compared to language-based educational interventions. In selective attention, one study did identify a near-significant improvement in preschoolers with music training (Bowmer et al., 2018). We hypothesize that due to the relationship between selective attention and inhibition, music education might have an effect in this EF domain. However, only four studies evaluated selective attention independently.

We also observed that orchestra-based interventional studies were unable to identify an improvement in EF task performance. However, these studies included school-age children without prior music education (Alemán et al., 2017; Sachs et al., 2017; Hennessy et al., 2019). Failure to identify an effect of music training may be explained by the age-dependent effect that was previously hypothesized. In contrast, some case-control studies did identify an association between prior music training and EF performance (Degé et al., 2011; Bayanova et al., 2022; Chen et al., 2022). This raises the question of whether RCTs in school-age children are the ideal study design to evaluate an effect that is probably acquired early in life. Perhaps further RCTs evaluating music training in preschool children with a long follow-up will be able to determine the effect of early music training.

The challenges of clinical research on music education and child development have been addressed by several authors. Of note, the definition of music education can be widely variable and as we observed in this review, there are several variables that are not standardized. Some examples include the amount of training (“dosage-effect”) and the time of follow-up. Moreover, the evaluation of music education also brings up philosophical and political questions that, although not within the scope of this article, are worth mentioning. What is the role of music education in a child's life, and which are the reasons to encourage music training, if any? What should be the approach to advocating for music education on a political ground where it can be interpreted as part of a liberal agenda? (Ilari, 2020). Studies evaluating the effects of music education in child development will continue to raise these questions for discussion.

This review has some limitations that should be addressed as well. Only two blinded authors participated in the selection process and we used three databases to retrieve relevant articles. However, we consider that most of the literature on this topic was evaluated in our selection process given that this is a highly specific subject. We also used appropriate tools to critically appraise the studies retrieved in the search and presented all the results with the most relevant information summarized in Table 4.

Through this study, we can conclude that the available evidence suggests a beneficial effect of early music training in the development of EFs, particularly inhibitory control, and to a lesser extent, working memory and cognitive flexibility. The size of this effect and the role of age are questions that can only be solved through further research. However, studies that evaluated music education among preschoolers were more likely to identify a significant effect in executive performance compared to older children. Active processes of maturation and neurodevelopment may explain why younger children are more susceptible to the effects of music training (Rauscher and Hinton, 2006).

Additionally, active music interventions appear to be more strongly associated with EF development in older children. Interestingly, interventions in preschoolers were mostly non-instrumental and yet several studies found a significant effect of music training in this population. These mixed results reflect the complexity of music training and may encourage hypotheses aiming to compare the effects of different music education techniques in cognitive development. Through this systematic review we also aim to lighten the interest in studying the impact of music education in neurodevelopment and executive performance.

## Data availability statement

The original contributions presented in the study are included in the article, further inquiries can be directed to the corresponding author.

## Author contributions

DR-G and CT-G conceived and designed the systematic review, performed the critical appraisal and full-text selection of the articles, filtered the articles by title and abstract, and read and edited the initial draft of the manuscript. DR-G performed the search and was responsible for writing the initial draft of the manuscript. All authors read and approved the final version of this manuscript.

## Conflict of interest

The authors declare that the research was conducted in the absence of any commercial or financial relationships that could be construed as a potential conflict of interest.

## Publisher's note

All claims expressed in this article are solely those of the authors and do not necessarily represent those of their affiliated organizations, or those of the publisher, the editors and the reviewers. Any product that may be evaluated in this article, or claim that may be made by its manufacturer, is not guaranteed or endorsed by the publisher.
